# Clinical features of treatment-naive patients with hepatitis B virus infection: A community-based survey from high- and intermediate-hepatitis B endemicity regions in Southeast China

**DOI:** 10.1097/MD.0000000000006660

**Published:** 2017-04-21

**Authors:** Wei Wu, Yu Zhu, Chenbo Yu, Shigui Yang, Bing Ruan, Yu Chen, Lanjuan Li

**Affiliations:** a State Key Laboratory for Diagnosis and Treatment of Infectious Disease, The First Affiliated Hospital, School of Medicine, Zhejiang University; b Collaborative Innovation Center for Diagnosis and Treatment of Infectious Diseases, Hangzhou, Zhejiang, People's Republic of China.

**Keywords:** clinical feature, community-based study, hepatitis B, Southeast China, treatment naïve

## Abstract

The aim of study was to investigate the clinical features of treatment-naive patients in 2 regions with high- and intermediate-hepatitis B endemicity level in Southeast China and provide the baseline data for monitoring health or planning therapy.

This study included 8207 cases of treatment-naive patients with hepatitis B virus (HBV) infection from Yuhuan (YH, high-hepatitis B endemicity region) and Shaoxing (SX, intermediate-hepatitis B endemicity region) during 2014–2015. Clinical data were collected from the patients. Blood samples were kept for detecting hepatitis B surface antigen, hepatitis B envelope antigen (HBeAg), hepatitis B envelope antibody, hepatitis B surface antibody, hepatitis B core antibody, liver function, HBV deoxyribonucleic acid, and alpha-fetoprotein. All persons underwent B ultrasound to exclude liver cirrhosis or cancer.

Of all 8207 HBsAg-positive patients, 52.9% patients were in the low-replication (LR) stage and 30.3% in the HBeAg-negative chronic hepatitis B (ENH) stage; 8.8% cases were in the ENH stage with elevated alanine aminotransferase (ALT). More male than female patients were in immune clearance (IC) or ENH stages with elevated ALT (10.4% vs 4.8%, 12.1% vs 5.3%, respectively, *P* < .05). The percentage of patients in IC and immune tolerant (IT) stages declined with increasing age, whereas the percentages of ENH with elevated ALT stage were highest in 40 to 60 years.

The percentage of patients in IT and IC stages was higher in YH than in SX (9.4% vs 3.8%, 9.9% vs 4.2%, respectively, *P* < .05). More patients had HBVDNA≥10^5^ IU/mL in YH than in SX (24.6% vs 16.0%, *P* < .05), and more male than female patients had HBVDNA≥10^5^ IU/mL(24.5% vs 17.9%, *P* < .05).

Clinical features varied in treatment-naive patients with HBV infection between different genders and regions. More attention should be paid to the surveillance and therapy of patients in YH especially male patients for the prevention and prognosis of hepatitis B.

## Abstract 2

Supplemental Digital Content is available in the text

## 1 Introduction

Hepatitis B virus (HBV) infection remains a major public health problem worldwide, especially in China.^[1–3]^ With the economic development, prophylaxes of HBV vaccine and antiviral therapy in China have increased in recent years, changing the spectrum of HBV infection. Moreover, the understanding of the clinical features and natural history of this disease has improved, facilitating efficient treatment of patients. However, most of these data are from hospitals or clinics; data from communities are sparse.

From 2009 to 2010, a community-based epidemiological study was conducted in 12 counties of Zhejiang Province, Southeast China, to investigate the epidemiology of HBV in adults and provide the most recent baseline data for HBV prevention.^[4]^ A total of 761,544 residents of 12 counties were recruited according to their location, population density, and economic development. HBV surface antigen (HBsAg) and alanine amino transferase (ALT) were determined. The result showed that the standardized HBsAg carrier rate was 6.13% overall; however, the obvious regional disparity of HBV epidemiology existed. The HBsAg carrier rate was 14.62% among fishermen living in coastal regions, which was significantly higher than that in nonfishermen (7.07%) living in plains. The HBsAg positivity rate varied from 8.16% in male participants to 6.36% in female participants (*P* < .01). Two of the aforementioned 12 counties, Yuhuan (YH) and Shaoxing (SX), had different HBsAg positivity rates. The HBsAg carrier rate was 11.1% in YH and 6.6% in SX (*P* < .001).^[5]^ The world can be broadly classified into high (HBsAg prevalence >8%), intermediate (HBsAg prevalence 2%–8%), and low (HBsAg prevalence <2%) HBV endemicity regions.^[6]^ YH has been classified as a high-HBV endemicity region and SX as an intermediate-HBV endemicity region.

YH and SX are all located in Zhejiang Province, south of Yangtze River Delta in Southeast China. The distance between the 2 regions is about 280 km. Yuhuan is an island region, and Shaoxing is in plains. The population of YH and SX is 425.5 thousand and 443.0 thousand, respectively. The gross domestic product per capita of YH and SX was nearly 101,961 RMB ($16,370) and 90,127 RMB ($14,470) in 2015. Nearly 50% of the population in YH comprised fishermen or people involved in fishery-related occupations, whereas most people in SX were farmers or workers. The definite reason for the disparity in the HBV epidemiology of the 2 regions remains unknown. The clinical features of treatment-naive patients with HBV infection from different HBV endemicity regions were unclear either.

Based on the former studies, a community-based survey of clinical features of treatment-naive patients with HBV infection in Yuhuan and Shaoxing was conducted from 2014 to 2015. The purpose of this study was to demonstrate the clinical features of HBV infection from the overall population of the community, including the proportion of hepatitis B envelope antigen (HBeAg)-positive/negative hepatitis, HBV deoxyribonucleic acid (DNA) positive ratio, distribution of clinical stages related to age or gender, and proportion of candidates for anti-HBV treatment. The results might help one in understanding the spectrum of HBV infection nowadays or providing the data to evaluate the duration or cost of antiviral therapy, or knowing which population needed closer follow-up. The second aim of the study was to compare the clinical features of treatment-naive patients with HBV infection from YH and SX, classified as high- and intermediate-HBV endemicity regions, respectively. The current prophylaxis and treatment strategies were uniform and did not consider the HBV endemicity level in China. The results might help in improving the healthcare system for different HBV endemicity regions with different clinical features.

## 2 Methods

### 2.1 Criteria and process for admission and exclusion

All residents of Zhejiang Province, including SX and YH regions, were provided the plan for health examination every 2 years free of charge since 2005. The plan included a physical examination and routine laboratory testing. Since 2010, HBsAg test and ALT assays were added to the plan in 12 countries of the province including the above 2 regions with patients’ consent.

Based on the results of former health examination, a community-based survey was conducted in YH and SX regions from 2014 to 2015. The towns/subdistricts in each region were divided into 3 different levels according to the economic status. In each level, 1 village/residents’ committees was randomly selected. Finally, 3 communities (Qianqing, Qixian, and Keqiao) from SX and 3 communities (Chengguan, Chumen, and Kanmen) from YH were selected. Permanent residents, aged 20 years or older, who had lived in the aforementioned communities for more than 0.5 years, were eligible for enrollment. Both urban and rural populations were included. All these patients were HBsAg positive for more than 6 months.

**Figure 1:**
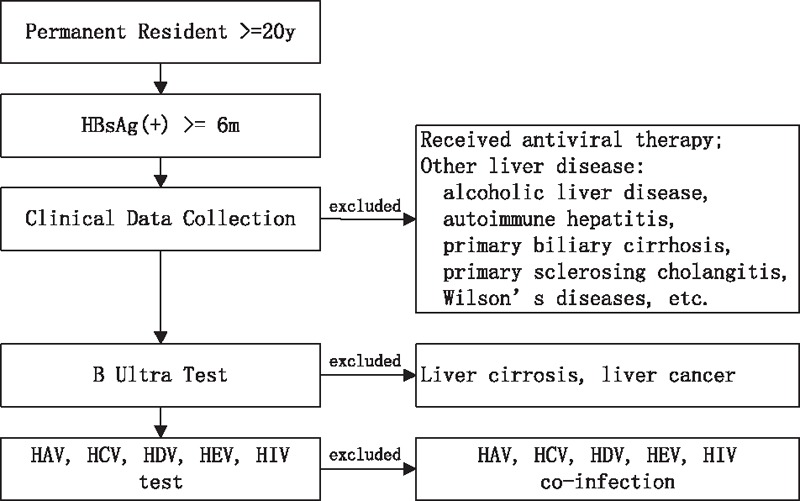
Process of enrollment and exclusion. HAV = hepatitis A virus, HBsAg = hepatitis B surface antigen, HCV = hepatitis C virus, HDV = hepatitis D virus, HEV = hepatitis E virus, HIV = human immunodeficiency virus.

Clinical data were collected by community doctors. A list of patients who were HBsAg positive for more than 6 months was established in the above 6 communities. The community doctors contacted these patients by telephone, leaflet, or letters, and asked them to undergo further examination. Data were recorded including symptoms, signs, medicine history, family history, and side effects. All the patients who had received antiviral therapies such as interferon or nucleoside/nucleotide analogs were excluded. Those who were found to have other liver diseases, including alcoholic liver disease, nonalcoholic fatty liver, autoimmune hepatitis, primary biliary cirrhosis, primary sclerosing cholangitis, and Wilson's disease, were also excluded. Liver ultrasound was conducted by 2 senior doctors from the radiology department of each community health center to screen hepatic carcinoma and liver cirrhosis. Patients with hepatitis A virus (HAV), hepatitis C virus (HCV), hepatitis D virus (HDV), hepatitis E virus (HEV), or human immunodeficiency virus (HIV) coinfection were excluded (Fig. 1).

This study was approved by the ethics committee of the First Affiliated Hospital of Medicine, Zhejiang University. All participants provided informed consent. The data regarding age, gender, or occupation were unveiled with patients’ consent.

### 2.2 Serological testing

Approximately 10 mL of venous blood was collected from each participant. Name, age, sex, district, and so on were recorded on standardized information forms. Blood samples were kept in a cold container and immediately transferred to ADICON Clinical Laboratories, Inc., as reported,^[7]^ where sera were separated and stored at –70°C until processing. ADICON Clinical Laboratories, Inc., which is located in Hangzhou, carried out the serological assessment in accordance with the manufactures’ instructions.

All samples were tested for HBV surface antigen (HBsAg), HBV e antigen (HBeAg), HBV core antibody (anti-HBc), HBV envelope antibody (anti-HBe), and HBV surface antibody (anti-HBs) using enzyme immunoassay kits (ACON/Architect i2000S, Hangzhou, China). Liver functions were tested including ALT, aspartate transaminase, total bilirubin, direct bilirubin, and so forth, by ADICON Co. Ltd, Hangzhou, China. The upper limit of normal (ULN) of ALT was 38IU/L. Serum HBV DNA was quantified using the real-time fluorescence quantitative polymerase chain reaction machine (Autrax/Slan, Shanghai,China) with a detection range of 10^3^IU/mL to 10^8^ IU/mL.^[7]^ For patients with HBVDNA>10^8^ IU/mL, the HBVDNA assay was repeated after a dilution of 1:1000. Alpha-fetoprotein (AFP) levels were determined by an AFP EIA Test Kit (BoSai, Zhengzhou, China). Antibodies of HAV, HCV, HDV (Wangtai, Beijing, China), HEV, or HIV (Lizhu, Zhuhai, China) were tested using enzyme immunoassay kits.

### 2.3 Stages of persistent hepatitis B virus infection

According to the European Association for the Study of the Liver (EASL) (2012)^[8]^ and American Association for the Study of Liver Diseases (AASLD) (2016)^[9]^ clinical practice guidelines for chronic HBV infection, the natural history of chronic HBV infection can be divided into 4 stages: the immunotolerant (IT) stage, the immune reactive HBeAg-positive stage (or immune clearance, IC), the inactive HBV carrier stage (or low-replication, LR), and the HBeAg-negative chronic hepatitis B (ENH) stage. The IT phase is characterized by HBeAg positivity, high levels of HBV replication, normal or low levels of amintransferases, mild or no liver necroinflammation and no or slow progression of fibrosis. Patients in this phase are highly contagious. The IC phase is characterized by HBeAg positivity, elevate (≥20,000 IU/mL) HBV DNA levels, increased or fluctuating levels of amintransferases, moderate or severe liver necroinflammation and more rapid progression of fibrosis. The LR phase is characterized by HBeAg negative, very low or undetectable serum HBV DNA levels (<2000 IU/mL) and normal amintransferases. The ENH phase is characterized by HBeAg negative, periodic reaction with a pattern of fluctuating levels of HBV DNA (≥2000 IU/mL)and amintransferases and active hepatitis.

Some adjustments were made to the aforementioned definition, and LR and ENH stages were described as subgroups with normal or elevated ALT to include most patients in the present study and describe the profile of chronic HBV infection in detail (Table 1).

**Table 1:**
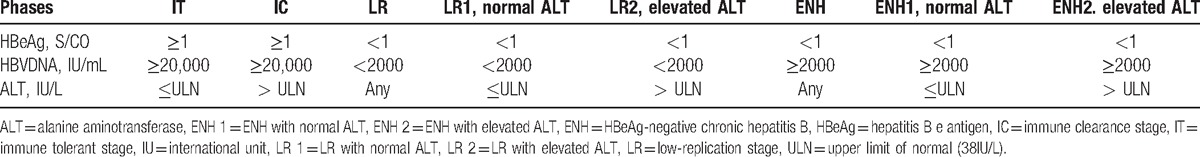
Definitions of phases of persistent hepatitis B virus infection.

### 2.4 Data analysis

Data were managed and analyzed using SPSS 19.0 (SPSS, Chicago, IL). Continuous variables are presented as mean ± SD, or as median (quartile range) if variables are not normal distributed. Categorical variables are presented as numbers and percentages, and the chi-square test was used for statistical analysis. All tests were 2-sided, and *P* value less than .05 was considered statistically significant. The Bonferroni correction method was used for multiple significance tests.

## 3 Results

### 3.1 Enrollment and exclusion

A total of 10,834 patients were agreed to enter into the process of enrollment and exclusion, and finally 8207 patients of hepatitis B infection were included in the present study. Among them, 3151 patients were from SX and 5056 patients from YH; 4239 patients were males, and 3968 were females. The average age is 48.07 ± 12.10 years. All the patients were divided into 3 age groups as 20 to <40 years, 40 to 60 years and >60 years old.

### 3.2 Clinical stages of persistent hepatitis B virus infection in the 2 regions

A total of 8207 patients were analyzed for the stages of persistent HBV infection (Table 2). Of all 8207 HBsAg-positive patients, the LR stage (52.9%) was the most common stage. Among them, 45.3% were in the LR stage with normal ALT and 7.6% cases were in the LR stage with elevated ALT. Also, 30.3% cases were in the ENH stage, 21.5% were in the ENH stage with normal ALT, and 8.8% were in the ENH stage with elevated ALT. The percentages of IT and IC were 7.3% and 7.7%, respectively.

**Table 2:**
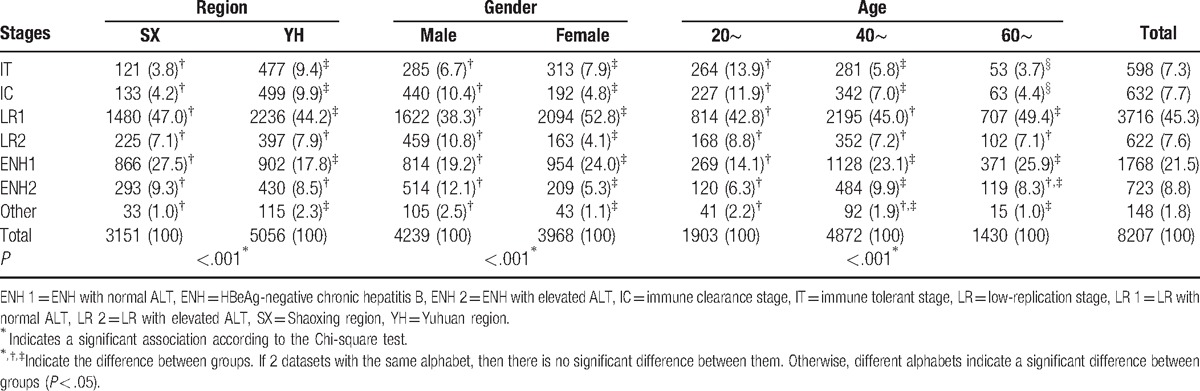
Distribution of clinical stages of persistent hepatitis B virus infection among region, gender, and age groups.

**Figure 2:**
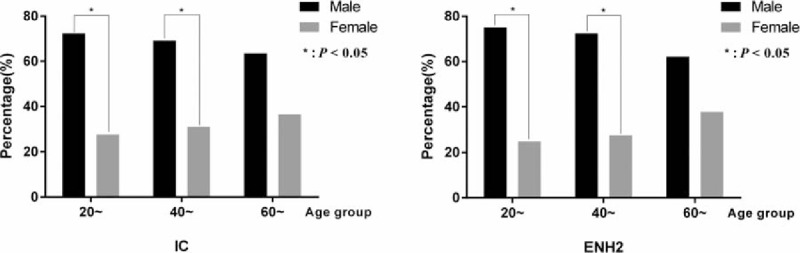
Percentages of patients in IC and ENH stages with elevated ALT in male and female groups. ALT = alanine aminotransferase, ENH 2 = ENH with elevated ALT, IC = immune clearance stage.

A comparison of the difference in clinical stages between male and female showed that the percentages of patients in IC and ENH stage with elevated ALT in male were higher than female (10.4% vs 4.8%, 12.1% vs 5.3%, respectively, *P* < .05), but the percentages in IT and ENH stage with normal ALT in female were higher than male (7.9% vs 6.7%, 24.0% vs 19.2%, respectively, *P* < .05). Patients in IC and ENH stages with elevated ALT may be the candidates for anti-HBV treatment.^[8]^ The detailed percentages of every clinical stage from male and female groups in the 2 regions (Supplementary Table 1) showed that more male than female patients were observed in the IC or ENH stage with elevated ALT in the age groups of 20 to 40 years (Fig. 2).

The data in Table 2 also showed that with the increasing age, the percentage of patients in the IC and IT stage declined (*P* < .05). The percentages of patients in the ENH stage with elevated ALT were the highest in the age groups of 40 to 60 years.

A comparison of the difference in clinical stages between SX and YH showed that the percentage of patients in IT and IC stages in YH were higher than the percentage of patients in the SX stage (9.4% vs 3.8%, 9.9% vs 4.2%, respectively, *P* < .05). Patients in IT and IC stages were HBeAg positive, which meant more patients were HBeAg positive in YH than in SX. The percentages of patients in ENH with normal ALT were higher in SX than in YH (27.5% vs 17.8%, *P* < .05). The detailed distribution of clinical stages of persistent hepatitis B virus infection between SX and YH regions was shown in Supplementary Table 2.

### 3.3 Distribution of HBV DNA level in the 2 regions

**Figure 3:**
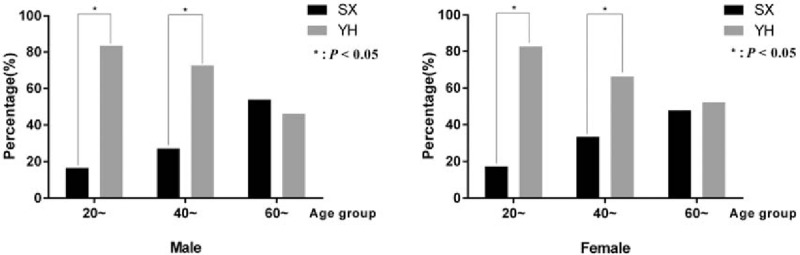
Percentages of patients with HBVDNA ≥105 IU/mL in SX and YH regions. DNA = deoxyribonucleic acid, SX = Shaoxing region, YH = Yuhuan region.

The HBVDNA level was classified into 3 levels: <2000 IU/mL as the low level, 2000∼ 10^5^ IU/mL as the medium level, and ≥10^5^ as the high level (Table 3). The percentage of HBV DNA negative patients, which meant their HBV DNA<2000 IU/mL were 54.0% totally. More patients had HBVDNA≥10^5^ IU/mL in YH than in SX (24.6% vs 16.0%, *P* <.05). The Supplementary Table 3 of distribution of HBV DNA level between SX and YH regions showed that more patients had HBVDNA≥10^5^ IU/mL in YH than in SX in both females and males of 20∼ and 40∼ age groups (Fig. 3).

**Table 3:**
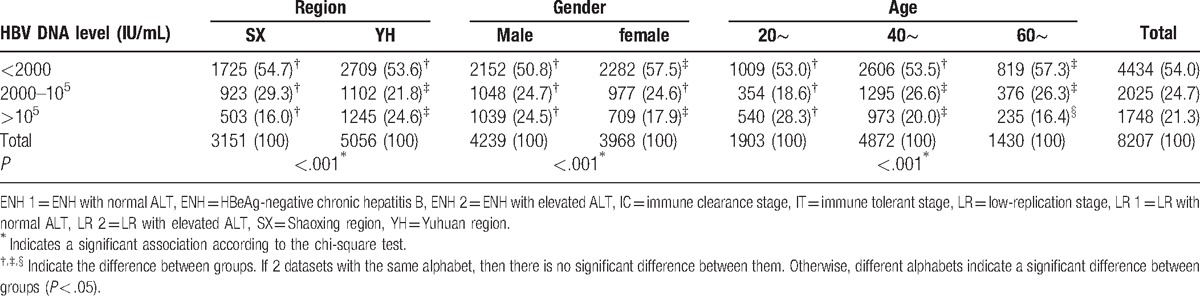
Distribution of HBV DNA level among region, gender, and age groups.

More male than female patients had HBVDNA≥10^5^ IU/mL(24.5% vs 17.9%, *P* < .05). The Supplementary Table 4 of distribution of HBV DNA level between male and female groups showed that more male than female had HBVDNA≥10^5^ IU/mL in SX of all age groups, but only 40∼ age groups in the YH region. The percentage of patients with HBVDNA≥10^5^ IU/mL decreased with increasing age (*P* < .05).

## 4 Discussion

HBV infection is a significant public health problem that may lead to chronic liver disease, cirrhosis, and hepatocellular carcinoma (HCC).^[10]^ Generally, exposure to HBV can cause a broad spectrum of infections. Around 90% to 95% of HBV infection in adults develops into acute infection followed by viral clearance, whereas 5% to 10% develop into chronic infection.^[11–13]^ HBV vaccination has been extended most of the infants in China and dramatically decreased the HBsAg positive rate. Today, the HBsAg positive rate of child younger than 5 years is 0.32% only. However, there are still 80 million HBV carrier in China. People usually lack the awareness of this chronic disease, especially in rural regions. Quite a few people did not realize that they had persistent HBV infection until they had symptoms such as fatigue, jaundice, or even ascites. From 2005, all residents of Zhejiang Province were provided the plan for health examination every 2 years free of charge. Since 2010, HBsAg test and ALT assays, and B ultrasound in some parts of the region were added to the plan. However, the data were not enough for the diagnosis of clinical stages of hepatitis B, or to follow up the patients.

The purpose of our study was to demonstrate the clinical features of treatment-naive patients with HBV infection from the overall population of the community. The present study showed that 52.9% cases of these HBsAg-positive patients were in the LR stage and 30.3% in the ENH stage. Moreover, 8.8% cases were in the ENH stage with elevated ALT. Since this study was a cross-sectional study, it did not take into account the changes or fluctuation in viral load and ALT level over time. Besides, some patients usually took the traditional Chinese medicine to reduce the ALT level. The EASL guideline also pointed out that it was important and sometimes difficult to distinguish true inactive HBV carriers from patients with active HBeAg-negative hepatitis. A minimum follow-up of 1 year with ALT levels at least every 3 to 4 months and serum HBV DNA levels is required before classifying a patient into a specific stage. So, a close follow-up would show more patients in the ENH stage with elevated ALT.

As reported earlier, the percentage of HBeAg-negative hepatitis B increased owing to some reasons such as the prophylaxis of HBV vaccine, decrease in acute HBV infection, aging of the population with HBV infection, and popular use of antiviral therapy in recent years.^[14]^ The treatment for HBeAg-negative hepatitis has its own indications and duration. If HBeAg-positive hepatitis is treated with nucleoside/nucleotide analogues (NAs), finite duration is achievable with anti-HBe seroconversion. However, long-term or infinite treatment with NAs is necessary for patients with HBeAg-negative hepatitis,^[15–17]^ which means more viral mutations and increased cost for these patients.

The percentage of patients in IC and IT stages declined with increasing age; however, the percentages of patients in the ENH stage with elevated ALT were the highest in the age groups of 40 to 60 years in the 2 regions. A study showed that in HBeAg-negative hepatitis, the cumulative progression rate from the inactive carrier state to chronic hepatitis B was as high as 24% after 4 years.^[18]^ In HBeAg-negative hepatitis, the older age groups had significantly higher proportions of patients with active disease. These findings suggested that even for patients who undergo HBeAg seroconversion, continual monitoring is mandatory. Meanwhile, HBV infection in elderly patients is usually accompanied by other diseases, which increases the difficulties encountered during therapy, such as the drug–drug interaction or insufficiency of liver or kidney function.

This study also found that more male than female patients were observed in the IC or ENH stage with elevated ALT(10.4% vs 4.8%, 12.1% vs 5.3%, respectively, *P* < .05). Since the EASL guidelines recommended that the criteria for antiviral therapy were patients with HBV DNA levels above 2000 IU/mL, serum ALT levels above ULN, and severity of liver disease assessed by liver biopsy, patients in IC and ENH stages with elevated ALT in the present study might be the candidates for anti-HBV treatment. So, our results showed that more male patients in this study might need antiviral therapy than female. In the AASLD guideline, the indications for antiviral therapy included an elevation of ALT >2ULN or evidence of significant histological disease plus elevated HBV DNA above 2000 IU/mL (HBeAg negative) or above 20,000 IU/mL (HBeAg positive). The ULN for ALT in healthy adults is 30 U/L for males and 19U/L for females. In China, the same ULN of ALT is still used for males and females. Further study would be needed whether to use different ULN for ALT in male and female groups in China.

A comparison of the difference in clinical stages in SX and YH showed that the percentage of patients in IT and IC stages were higher in YH than in SX (9.4% vs 3.8%, 9.9% vs 4.2%, respectively, *P* < .05). More patients had HBVDNA≥10^5^ IU/mL in YH than in SX (24.6% vs 16.0%, *P* < .05). More male patients with HBVDNA≥10^5^ IU/mL level than female (24.5% vs 17.9%, *P* < .05). The mechanism underlying the difference between the 2 regions and genders remains unknown. HBV infection may be considered as a complex trait with viral, environmental, and genetic components.^[19]^ Under the pressure of a certain environmental factor such as host genetic makeup or immunity system, the HBV virus may have some genetic variants to fit its microenvironment. Some mutations in the viral polymerase gene and its regulatory genes may result in nucleotide changes while overlapping with surface genes, which in turn may lead to changes in not only the replicating capability of HBV, but also the capability of viral assembly, secretion, and infectivity.^[20,21]^ Some relationship may exist among HbsAg-positive rate, HBeAg-positive rate, and HBV DNA titer.

Considering the difference in clinical features of HBV infection between regions and genders, more strategies should be implemented to reduce HBV spread in YH especially in male groups with the higher HBV DNA level. Currently, only the routine infant immunization and partially time-limited catch-up strategies for high risk groups were supported by government. The alternative strategies we suggested: First, the government should put more effort to improve public awareness for the spread of hepatitis B, since in our survey, it was found that the public awareness of hepatitis B is quite low (usually less than 20%). Since HBV is spread predominantly by percutaneous or mucosal exposure to infected blood and various body fluids, including saliva, menstrual, vaginal, and seminal fluids,^[22,23]^ people in YH and male group should know how to avoid high-risk behaviors, such as unsafe sexual behavior, sharing razors, intravenous and percutaneous drug abuse, use of inadequately sterilized syringes and needles, tattooing, body piercing, and acupuncture, and so forth. Second, besides the routine infant immunization, time-limited catch-up strategies targeted at unvaccinated people in the older age groups may be needed in groups with the higher HbsAg-positive rate to hasten the development of population-based immunity and decrease more rapidly the incidence of acute hepatitis B;^[24,25]^ Since most of these vaccination is not supported by government nowadays, more funds in needed in the inoculation in future. Third, since perinatal transmission is still the major route of HBV transmission particularly in China,^[26]^ mothers with very high concentrations of HBV DNA should receive antiviral therapy in pregnancy, despite both HBV vaccination and HBIG prophylaxis.^[27,28]^ For example, a national project of prenatal transmission block of hepatitis B was carried out in China since 2015. The project included 2 stages, first, 1000 cases of HBsAg pregnancy were registered and followed up in 10 university teaching hospital. Second, a network of preventing HBV mother-to-child transmission would be gradually set up in China. These experiences can be popularized in high HBV endemic areas such as YH.

This study had several limitations. First, this study was a cross-sectional study; therefore, it did not take into account the changes or fluctuation in viral load and ALT level over time. Second, the study excluded patients younger than 20 years old, or those with decompensate liver disease, cirrhosis, or HCC. Third, since all patients were from communities, liver biopsy was not performed routinely. However, liver biopsy is important in patients with elevated HBV DNA and ALT levels, which are not high enough to start antivirus treatment immediately.

In conclusion, clinical features varied in treatment-naive patients with HBV infection between different genders and regions. More attention should be paid to the surveillance and therapy of patients in YH especially male patients for the prevention and prognosis of hepatitis B.

## Supplementary Material

Supplemental Digital Content
