# Supplementary material for: Clinical features of treatment-naive patients with hepatitis B virus infection: A community-based survey from high- and intermediate-hepatitis B endemicity regions in Southeast China
Source: Medicine (Baltimore). 2017 Apr 21;96(16):e6660. doi: 10.1097/MD.0000000000006660 (PMC5406089; doi:10.1097/MD.0000000000006660)
Supplement: Supplemental Digital Content [file medi-96-e6660-s001.doc]

Supplementary table 1 Distribution of clinical stages of persistent hepatitis B virus infection between male and female groups.

| stages | SX, 20~(n=499) | | YH, 20~(n=1404) | | SX, 40~(n=1870) | | YH, 40~(n=3004) | | SX,60~(n=782) | | YH, 60~(n=648) | |
| --- | --- | --- | --- | --- | --- | --- | --- | --- | --- | --- | --- | --- |
|  | male(n=232) | female(n=267) | male(n=801) | female(n=603) | male(n=799) | female(n=1071) | male(n=1625) | female(n=1379) | male(n=430) | female(n=352) | male(n=352) | female(n=296) |
| IT | 16(6.9)_a_ | 24(9.0)_a_ | 104(13.0)_a_ | 120(19.9)_b_ | 24(3.0)_a_ | 36(3.4)_a_ | 104(6.4)_a_ | 117(8.5)_b_ | 16(3.7)_a_ | 5(1.4)_b_ | 21(6.0)_a_ | 11(3.7)_a_ |
| IC | 29(12.5)_a_ | 10(3.7)_b_ | 135(16.9)_a_ | 53(8.8)_b_ | 41(5.1)_a_ | 26(2.4)_b_ | 195(12.0)_a_ | 80(5.8)_b_ | 17(4.0)_a_ | 10(2.8)_a_ | 23(6.5)_a_ | 13(4.4)_a_ |
| LR1 | 80(34.5)_a_ | 160(59.9)_b_ | 278(34.7)_a_ | 296(49.1)_b_ | 298(37.3)_a_ | 563(52.6)_b_ | 595(36.6)_a_ | 739(53.6)_b_ | 198(46.0)_a_ | 181(51.4)_a_ | 173(49.1)_a_ | 155(52.4)_a_ |
| LR2 | 33(14.2)_a_ | 17(6.4)_b_ | 102(12.7)_a_ | 16(2.7)_b_ | 80(10.0)_a_ | 46(4.3)_b_ | 179(11.0)_a_ | 47(3.4)_b_ | 36(8.4)_a_ | 13(3.7)_b_ | 29(8.2)_a_ | 24(8.1)_a_ |
| ENH1 | 41(17.7)_a_ | 43(16.1)_a_ | 97(12.1)_a_ | 88(14.6)_a_ | 214(26.8)_a_ | 325(30.3)_a_ | 277(17.0)_a_ | 312(22.6)_b_ | 123(28.6)_a_ | 120(34.1)_a_ | 62(17.6)_a_ | 66(22.3)_a_ |
| ENH2 | 26(11.2)_a_ | 11(4.1)_b_ | 64(8.0)_a_ | 19(3.2)_b_ | 131(16.4)_a_ | 66(6.2)_b_ | 219(13.5)_a_ | 68(4.9)_b_ | 38(8.8)_a_ | 21(6.0)_a_ | 36(10.2)_a_ | 24(8.1)_a_ |
| other | 7(3.0)_a_ | 2(0.7)_a_ | 21(2.6)_a_ | 11(1.8)_a_ | 11(1.4)_a_ | 9(0.8)_a_ | 56(3.4)_a_ | 16(1.2)_b_ | 2(0.5)_a_ | 2(0.6)_a_ | 8(2.3)_a_ | 3(1.0)_a_ |

a, b, c indicate the difference between groups. If two datasets with the same alphabet, then there is no significant difference between them. Otherwise, different alphabets indicate a significant difference between groups (p<0.05)

ENH 1, ENH with normal ALT; ENH, HBeAg-negative chronic hepatitis B; ENH 2, ENH with elevated ALT; IC, immune clearance stage; IT, immune tolerant stage; LR, low-replication stage; LR 1, LR with normal ALT; LR 2, LR with elevated ALT; SX, Shaoxing region; YH, Yuhuan region.

supplementary table 2 Distribution of clinical stages of persistent hepatitis B virus infection between YH and SX

| stages | male, 20~(n=1033) | | female, 20~(n=870) | | male, 40~(n=2424) | | female, 40~(n=2450) | | male,60~(n=782) | | female, 60~(n=648) | |
| --- | --- | --- | --- | --- | --- | --- | --- | --- | --- | --- | --- | --- |
|  | SX(n=232) | YH(n=801) | SX(n=267) | YH(n=603) | SX(n=799) | YH(n=1625) | SX(n=1071) | YH(n=1379) | SX(n=430) | YH(n=352) | SX(n=352) | YH(n=296) |
| IT | 16(6.9)_a_ | 104(13.0)_b_ | 24(9.0)_a_ | 120(19.9)_b_ | 24(3.0)_a_ | 104(6.4)_b_ | 36(3.4)_a_ | 117(8.5)_b_ | 16(3.7)_a_ | 21(6.0)_a_ | 5(1.4)_a_ | 11(3.7)_a_ |
| IC | 29(12.5)_a_ | 135(16.9)_a_ | 10(3.7)_a_ | 53(8.8)_b_ | 41(5.1)_a_ | 195(12.0)_b_ | 26(2.4)_a_ | 80(5.8)_b_ | 17(4.0)_a_ | 23(6.5)_a_ | 10(2.8)_a_ | 13(4.4)_a_ |
| LR1 | 80(34.5)_a_ | 278(34.7)_a_ | 160(59.9)_a_ | 296(49.1)_b_ | 298(37.3)_a_ | 595(36.6)_a_ | 563(52.6)_a_ | 739(53.6)_a_ | 198(46.0)_a_ | 173(49.1)_a_ | 181(51.4)_a_ | 155(52.4)_a_ |
| LR2 | 33(14.2)_a_ | 102(12.7)_a_ | 17(6.4)_a_ | 16(2.7)_b_ | 80(10.0)_a_ | 179(11.0)_a_ | 46(4.3)_a_ | 47(3.4)_a_ | 36(8.4)_a_ | 29(8.2)_a_ | 13(3.7)_a_ | 24(8.1)_b_ |
| ENH1 | 41(17.7)_a_ | 97(12.1)_b_ | 43(16.1)_a_ | 88(14.6)_a_ | 214(26.8)_a_ | 277(17.0)_b_ | 325(30.3)_a_ | 312(22.6)_b_ | 123(28.6)_a_ | 62(17.6)_b_ | 120(34.1)_a_ | 66(22.3)_b_ |
| ENH2 | 26(11.2)_a_ | 64(8.0)_a_ | 11(4.1)_a_ | 19(3.2)_a_ | 131(16.4)_a_ | 219(13.5)_a_ | 66(6.2)_a_ | 68(4.9)_a_ | 38(8.8)_a_ | 36(10.2)_a_ | 21(6.0)_a_ | 24(8.1)_a_ |
| other | 7(3.0)_a_ | 21(2.6)_a_ | 2(0.7)_a_ | 11(1.8)_a_ | 11(1.4)_a_ | 56(3.4)_b_ | 9(0.8)_a_ | 16(1.2)_a_ | 2(0.5)_a_ | 8(2.3)_b_ | 2(0.6)_a_ | 3(1.0)_a_ |

a, b, c indicate the difference between groups. If two datasets with the same alphabet, then there is no significant difference between them. Otherwise, different alphabets indicate a significant difference between groups (p<0.05)

ENH 1, ENH with normal ALT; ENH, HBeAg-negative chronic hepatitis B; ENH 2, ENH with elevated ALT; IC, immune clearance stage; IT, immune tolerant stage; LR, low-replication stage; LR 1, LR with normal ALT; LR 2, LR with elevated ALT; SX, Shaoxing region; YH, Yuhuan region.

supplementary table 3 Distribution of HBV DNA level between SX and YH regions

| HBV DNA | male, 20~(n=1033) | | male, 40~(n=2424) | | male, 60~(n=782) | | female, 20~(n=870) | | female, 40~(n=2450) | | female, 60~(n=648) | |
| --- | --- | --- | --- | --- | --- | --- | --- | --- | --- | --- | --- | --- |
| level(IU/ml) | SX(n=232) | YH(n=801) | SX(n=799) | YH(n=1625) | SX(n=430) | YH(n=352) | SX(n=267) | YH(n=603) | SX(n=1071) | YH(n=1379) | SX(n=352) | YH(n=296) |
| <2000 | 118(50.9)_a_ | 396(49.4)_a_ | 385(48.2)_a_ | 810(49.8)_a_ | 235(54.7)_a_ | 208(59.1)_a_ | 179(67.0)_a_ | 316(52.4)_b_ | 613(57.2)_a_ | 798(57.9)_a_ | 195(55.4)_a_ | 181(61.1)_a_ |
| 2000~10^5^ | 62(26.7)_a_ | 142(17.7)_b_ | 255(31.9)_a_ | 393(24.2)_b_ | 118(27.4)_a_ | 78(22.2)_a_ | 49(18.4)_a_ | 101(16.7)_a_ | 326(30.4)_a_ | 321(23.3)_a_ | 113(32.1)_a_ | 67(22.6)_b_ |
| >10^5^ | 52(22.4)_a_ | 263(32.8)_b_ | 159(19.9)_a_ | 422(26.0)_b_ | 77(17.9)_a_ | 66(18.8)_a_ | 39(14.6)_a_ | 186(30.8)_b_ | 132(12.3)_a_ | 260(18.9)_b_ | 44(12.5)_a_ | 48(16.2)_a_ |

a, b, c indicate the difference between groups. If two datasets with the same alphabet, then there is no significant difference between them. Otherwise, different alphabets indicate a significant difference between groups (p<0.05)

ENH 1, ENH with normal ALT; ENH, HBeAg-negative chronic hepatitis B; ENH 2, ENH with elevated ALT; IC, immune clearance stage; IT, immune tolerant stage; LR, low-replication stage; LR 1, LR with normal ALT; LR 2, LR with elevated ALT; SX, Shaoxing region; YH, Yuhuan region.

supplementary table 4 Distribution of HBV DNA level between male and female groups

| HBV DNA | SX, 20~(n=499) | | SX, 40~(n=1870) | | SX, 60~(n=782) | | YH, 20~(n=1404) | | YH, 40~(n=3004) | | YH, 60~(n=648) | |
| --- | --- | --- | --- | --- | --- | --- | --- | --- | --- | --- | --- | --- |
| level(IU/ml) | male(n=232) | female(n=267) | male(n=799) | female(n=1071) | male(n=430) | female(n=352) | male(n=801) | female(n=603) | male(n=1625) | female(n=1379) | male(n=352) | female(n=296) |
| <2000 | 118(50.9)_a_ | 179(67.0)_b_ | 385(48.2)_a_ | 613(57.2)_b_ | 235(54.7)_a_ | 195(55.4)_a_ | 396(49.4)_a_ | 316(52.4)_a_ | 810(49.8)_a_ | 798(57.9)_b_ | 208(59.1)_a_ | 181(61.1)_a_ |
| 2000~10^5^ | 62(26.7)_a_ | 49(18.4)_b_ | 255(31.9)_a_ | 326(30.4)_a_ | 118(27.4)_a_ | 113(32.1)_a_ | 142(17.7)_a_ | 101(16.7)_a_ | 393(24.2)_a_ | 321(23.3)_a_ | 78(22.2)_a_ | 67(22.6)_a_ |
| >10^5^ | 52(22.4)_a_ | 39(14.6)_b_ | 159(19.9)_a_ | 132(12.3)_b_ | 77(17.9)_a_ | 44(12.5)_b_ | 263(32.8)_a_ | 186(30.8)_a_ | 422(26.0)_a_ | 260(18.9)_b_ | 66(18.8)_a_ | 48(16.2)_a_ |

a, b, c indicate the difference between groups. If two datasets with the same alphabet, then there is no significant difference between them. Otherwise, different alphabets indicate a significant difference between groups (p<0.05)

ENH 1, ENH with normal ALT; ENH, HBeAg-negative chronic hepatitis B; ENH 2, ENH with elevated ALT; IC, immune clearance stage; IT, immune tolerant stage; LR, low-replication stage; LR 1, LR with normal ALT; LR 2, LR with elevated ALT; SX, Shaoxing region; YH, Yuhuan region.
